# Cancer can be a purely epigenetic disorder

**DOI:** 10.7150/ijbs.109274

**Published:** 2025-01-06

**Authors:** Ya Meng, Heng Sun, Chu-Xia Deng

**Affiliations:** 1 Guangdong Provincial Key Laboratory of Tumor Interventional Diagnosis and Treatment, Zhuhai People's Hospital (The Affiliated Hospital of Beijing Institute of Technology, Zhuhai Clinical Medical College of Jinan University), Zhuhai, China.; 2 Cancer Center, Faculty of Health Sciences, University of Macau, Macau SAR, China.; 3 MOE Frontiers Science Center for Precision Oncology, University of Macau, Macau SAR, China.; 4 Zhuhai UM Science & Technology Research Institute, Hengqing, China.

It is widely acknowledged that tumors arise from mutations in oncogenes and tumor suppressor genes. These mutations endow tumor cells with capabilities such as uncontrolled proliferation, evasion of apoptosis, immune escape, and other hallmark characteristics, which are crucial for tumor initiation and progression. Recently, an intriguing study by V. Parreno *et al*. demonstrated that in *Drosophila melanogaster* (fruit fly), tumors can form without genetic mutations 1. Specifically, a 24-hour deficiency of the epigenetic regulator Polyhomeotic (*ph*), a component of Polycomb group (PcG) proteins, is sufficient to induce irreversible tumorigenesis. This finding challenges the prevailing notion that all tumors result from the accumulation of mutations and opens up new avenues for therapeutic strategies targeting these diseases.

The animal model in this study, *Drosophila*, has been extensively utilized in genetic and epigenetic research. Thomas Hunt Morgan's meticulous observation and analysis of how genetic traits are inherited in *Drosophila* led to the significant discovery that chromosomes play a crucial role in heredity 2. Additionally, Hermann Muller first identified in *Drosophila* that X-ray exposure causes DNA damage, leading to mutation generation and accumulation 3. Furthermore, a series of discoveries regarding the genetic and epigenetic regulation of early development in *Drosophila* have become foundational to developmental biology, offering insights applicable to other organisms, including humans. Importantly, approximately 75% of human disease-associated genes have homologs in *Drosophila*, making fruit flies an invaluable model for studying disease mechanisms, including cancer.

Interestingly, PcG proteins were initially identified in *Drosophila* as critical regulators of anterior-posterior patterning during early development 4. Subsequent research revealed that PcG proteins silence target genes via multiple cooperative epigenetic mechanisms. Importantly, further studies have shown that PcG proteins play a role in tumor suppression. For instance, dysfunction of PcG proteins, such as the *ph* null mutation, can induce tumor formation in *Drosophila* by activating oncogenic pathways like Notch and JAK-STAT 5. Moreover, PcG proteins in Drosophila contribute to transgenerational epigenetic inheritance, which refers to the transmission of functional states across generations without changes to the genome sequence 6. This suggests that cancerous states might be initiated, maintained, and potentially inherited through epigenetic rather than genetic mechanisms.

Indeed, in V. Parreno and colleagues' study 1, the transient loss of *ph* in the eye disc of *Drosophila* during the larval stage results in tumor formation in subsequent developmental stages (Figure 1). Notably, the re-expression of *ph* cannot reverse the tumor phenotype, despite the majority of affected signaling pathways controlled by *ph* being rescued. Further multi-omics analysis demonstrates that a few but critical irreversible transcriptional changes, epigenetic modifications, and chromatin accessibility alterations lead to the persistent activation of oncogenic pathways such as JNK/JAK-STAT. Additionally, through serial transplantation of allografts across multiple generations in *Drosophila*, epigenetically initiated cancers (EICs) are confirmed to be autonomous and immortal tumors.

As discussed, this study raises an intriguing question: could some human cancers be classified as epigenetic disorders? In certain cases, genetic and epigenetic abnormalities may collaborate in cancer initiation, with the latter potentially playing a more significant role in carcinogenesis. For instance, studies on ependymomas, a common pediatric brain tumor, have revealed very few cancer-driving mutations but a high frequency of CpG island methylation 7. Notably, the genes silenced by this methylation are exclusively PRC2 targets, suggesting that PRC2-associated epigenetic dysregulation might be a key driver in ependymomas. Furthermore, epigenetic dysregulation has been identified in multiple cancers, contributing to cancer initiation, metastasis, and the development of drug resistance.

A related important question is how to treat these EICs. As demonstrated in this study, re-establishing the transiently lost epigenetic regulator was ineffective. However, targeting downstream components of PRC1, such as *Stat92E* and/or *Zfh1* via RNAi, significantly reduced *ph*-dependent tumor growth, providing promising insights for future clinical applications. The irreversibility of EICs may be attributed to the timing of intervention. Specifically, resuming PH, *ph*-encoded protein expression might occur too late, missing the optimal therapeutic window. Therefore, the timing of treatment initiation is critical, as one non-negligible driver for the transient loss of *ph*-induced tumors is the intrinsic stemness of tumor-initiating cells. During early developmental stages, such as larval stages 1-3, eye disc cells retain the potential for proliferation and differentiation. Transient loss of *ph* or other PRC1 components alters the normal developmental trajectory. To some extent, short-term PRC1 dysfunction acts like a rail converter, guiding undifferentiated cells (the "speeding train") in the wrong direction. Similarly, previous studies have shown that transient activation of oncogenic signals, such as MYC and JNK, can induce tumorigenesis under specific conditions 8, 9. For instance, Pinal *et al.* reported that short-term activation of the JNK pathway in apoptosis-deficient cells can cause cancer in *Drosophila* 9. Thus, the type and state of cells in which oncogenic events occur are crucial for cancer initiation. This aligns with the "Bad Luck" theory of carcinogenesis proposed by Tomasetti and Vogelstein 10, which posits that higher cancer risk arises from mutation accumulation in more proliferative cells, such as stem cells.

One issue that warrants further consideration is the disparity between the macro- and micro-environments of cancers in *Drosophila*, rodent models, and human patients. The immune surveillance and regulation mechanisms in *Drosophila* are relatively limited. Despite possessing macrophage-like plasmatocytes, antimicrobial peptides, and other defense mechanisms, *Drosophila*'s overall immune response to cancer cells is significantly weaker compared to that in rodents and humans. It would be intriguing to investigate whether transient epigenetic dysfunction can initiate cancer in animal models with more robust immune systems. Overall, V. Parreno and colleagues' findings prompt a re-evaluation of cancer causality and intervention strategies. By conducting more in-depth fundamental research on cancer-initiating factors, drivers, and contributors using both animal models and clinical samples, and combining this with precise and personalized targeting approaches, we should hold greater promise for curing cancer in the future.

## Figures and Tables

**Figure 1:**
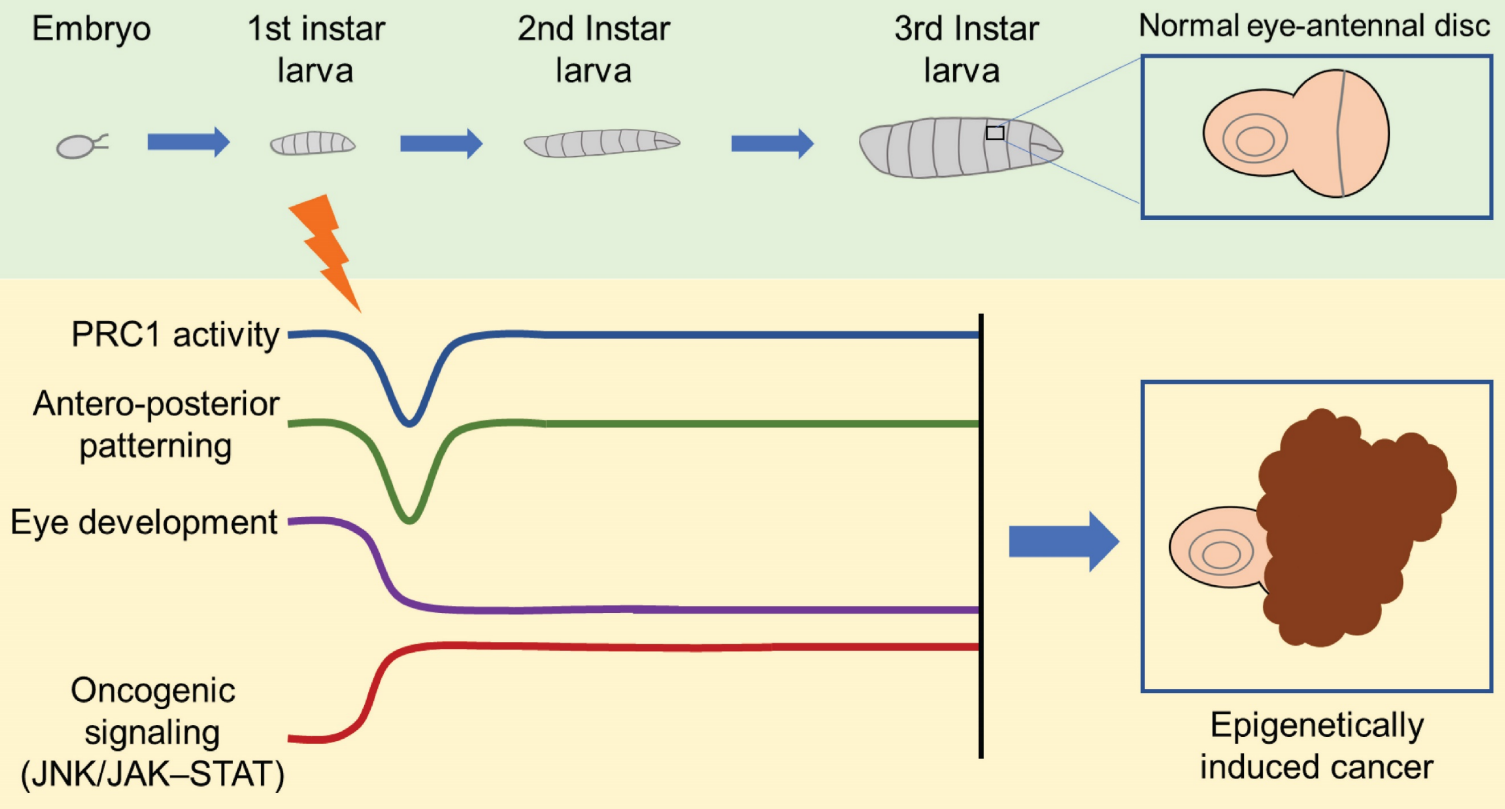
Transient *ph* knocking-down induces an epigenetic cancer fate. The transient loss of PRC1 component PH in eye disc of *Drosophila* at larva stage causes short-term and reversible dysfunction of PRC1 activity and antero-posterior patterning, while irreversible abnormality of eye development and persistent activation of oncogenic signaling like JNK/JAK-STAT, and eventually leads to cancer formation.
